# Secondary wound closure with a new transparent negative-pressure dressing

**DOI:** 10.1007/s00104-023-01864-3

**Published:** 2023-05-22

**Authors:** Gunnar Loske

**Affiliations:** Alfredstraße 9, 22087 Hamburg, Germany

**Keywords:** Vacuum therapy, Skin, Drainage, Film, Wound Care, Vakuumtherapie, Haut, Drainage, Folie, Wundmanagement

## Abstract

Secondary healing surgical wounds can be treated with negative-pressure therapy. Dressing changes can be painful due to the strong adherence of the polyurethane foam placed in the wound. After debridement and conditioning of the wound bed, secondary surgical wound closure with a surgical suture can be performed. Cutaneous negative-pressure therapy is used preventively after primary surgical suturing. Descriptions for secondary wound closure without a surgical suture are not known to date. The preparation and handling of an innovative transparent dressing for the cutaneous application of negative-pressure therapy is demonstrated here. The dressing assembly consists of a transparent drainage film and a transparent occlusion film. Negative pressure is applied via a tubing connector using a negative pressure pump. A new method of secondary wound closure using the transparent negative-pressure dressing is presented based on a case example. The treatment cycle with instructions for making the dressing is shown in a video.

**Video online:**

The online version of this article contains one video. The article and the video are online available (10.1007/s00104-023-01864-3). The video can be found in the article back matter as “Electronic Supplementary Material”.

## Abstract (de)

Sekundär heilende operative Wunden lassen sich mit der Unterdrucktherapie behandeln. Aufgrund der teilweise starken Adhärenz des in die Wunde eingelegten Polyurethanschaums können Verbandswechsel schmerzhaft sein. Nach der Konditionierung und dem Débridement des Wundgrunds kann der sekundäre Wundverschluss operativ mit einer chirurgischen Naht erfolgen. Die kutane Unterdrucktherapie wird auch präventiv nach primärer chirurgischer Naht angewendet. Beschreibungen für sekundäre Wundverschlüsse ohne eine chirurgische Naht sind bislang nicht bekannt. In der vorliegenden Arbeit werden die Fertigung und Handhabung eines innovativen transparenten Verbands für die kutane Anwendung der Unterdrucktherapie demonstriert. Die Verbandanordnung besteht aus einer transparenten Drainagefolie und einer transparenten Okklusionsfolie. Über eine Schlauchverbindung wird mit einer Unterdruckpumpe ein Unterdruck angelegt. Anhand eines Fallbeispiels wird eine neue Methode des sekundären Wundverschlusses unter Nutzung des transparenten Unterdruckverbands vorgestellt. Der Behandlungszyklus mit einer Anleitung zur Herstellung des Verbands wird in einem Video gezeigt.

Secondary healing surgical wounds can be treated with negative-pressure therapy. Dressing changes can be painful due to the occasionally strong adherence of the polyurethane foam placed in the wound. After debridement and conditioning of the wound bed, secondary surgical wound closure with a surgical suture can be performed. Cutaneous negative-pressure therapy is used preventively after primary surgical suturing [1–3].

Descriptions of secondary wound closure without a surgical suture are not known to date.

The preparation and handling of an innovative transparent dressing for the cutaneous application of negative-pressure therapy is demonstrated.

A case study is used to present a new method of secondary wound closure for postoperative wound healing by secondary intention using the transparent negative-pressure dressing (TNPD).

## Materials and methods

**Fig. 1:**
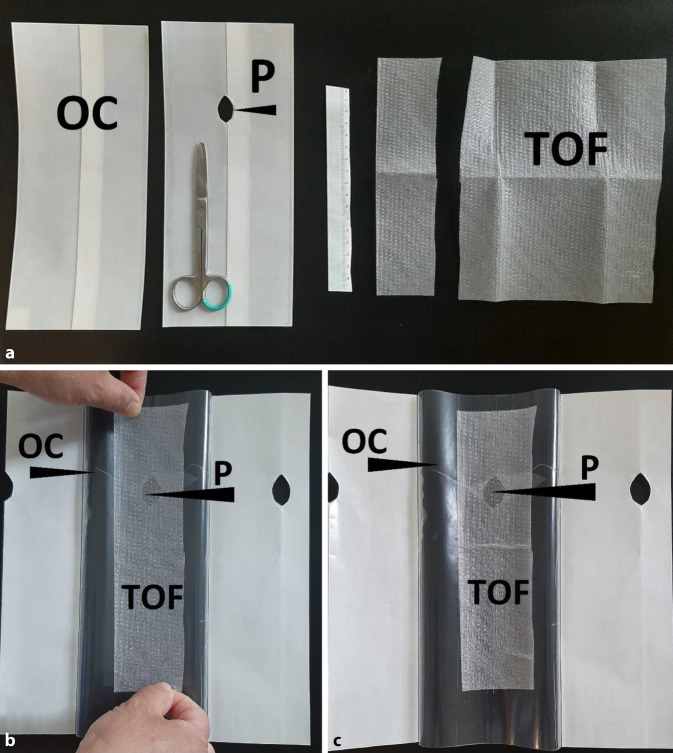
The material and method used to make a transparent negative-pressure dressing. **a** Transparent self-adhesive occlusion film (*OC*) (Suprasorb F, Lohmann & Rauscher International GmbH & Co. KG, Rengsdorf, Germany) with protective layer, transparent open-pore double-layer drainage film (*TOF*) (Suprasorb CNP Drainage Film, Lohmann & Rauscher International GmbH & Co. KG, Rengsdorf, Germany); cut an opening (*P*) in the OC with scissors; cut a strip of the TOF to the required size. **b** The TOF strip is placed on the adhesive side of the OC so that the TOF lies over the opening (*P*). **c** The finished dressing assembly, which can now be used to dress a wound. The TOF strip of the dressing is placed on the wound and fixed to the skin with the self-adhesive OC. Finally, a trackpad is glued over the P and tubing is used to connect it to the negative-pressure pump

The basic form of the TNPD (Fig. 1) is composed of the following materials:

- Transparent open-pore double-layer drainage film (TOF; Suprasorb CNP Drainage Film, Lohmann & Rauscher International GmbH & Co. KG, Rengsdorf, Germany)
- Transparent self-adhesive occlusion film (OC; Suprasorb F, Lohmann & Rauscher International GmbH & Co. KG, Rengsdorf, Germany; V.A.C. Drape, KCI USA, Inc., San Antonio, Texas, USA)
- Scissors

Scissors are used to cut the OC to size. The dimensions depend on the size of the wound; the OC should overlap the wound surface by several centimeters in all directions. Cut an opening (P) of about 2 cm in diameter in the middle. The tubing connector to the negative-pressure pump is subsequently attached on top.

A TOF strip approximately 4–5 cm wide is cut to the appropriate length for the wound.

The protective film of the self-adhesive OC is removed so that the adhesive surface is exposed. The TOF strip is attached to the adhesive side so that the P is covered with the TOF. With this last step, the TNPD dressing assembly is complete and ready for use.

**Fig. 2:**
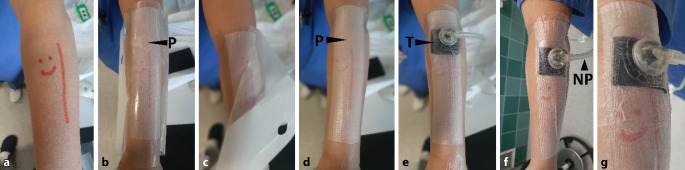
Application of the transparent negative-pressure dressing (TNPD) to a model wound. **a** Schematic representation of a superficial forearm wound. **b** The dressing assembly is applied to the wound using the open-pore side of the film. The occlusion film has an opening (*P*) through which the drainage film is exposed. **c** The protective film of the occlusion film is peeled off. **d** The dressing assembly is now adherent. **e** A trackpad (*T*) is attached above the opening P with a tubing connector, and T has an additional narrow polyurethane foam (Suprasorb CNP Wundschaum, Lohmann & Rauscher International GmbH & Co. KG, Rengsdorf, Germany) beneath it to facilitate suction. **f** Finally, a pump is used to apply negative pressure (*NP*) inside the trackpad tubing. **g** Close-up of the transparent dressing under negative pressure. The application of negative pressure causes the polyurethane foam to collapse and the occlusion film to be retracted over the pores of the drainage film. Due to the transparency of the TNPD, the wound and its environment can be assessed easily at any time during the application of negative pressure

The TNPD is applied using the same technique as for a standard adhesive dressing (Fig. 2). The side of the dressing with the TOF strip is placed on the wound and fixed to the skin with the self-adhesive OC. Finally, a self-adhesive tubing connector (trackpad) from a negative-pressure pump unit (ACTIV.A.C., KCI USA, Inc., San Antonio, Texas, USA; continuous suction −125 mm Hg) is attached to the surface of the TNPD over the P. The negative pressure is directed to the wound through a tubing connector via the TNPD. The TOF strip applies suction to the full surface of the wound and the skin directly near the wound (compression). Secretions are continuously and actively drained (drainage).

## Case study

**Fig. 3:**
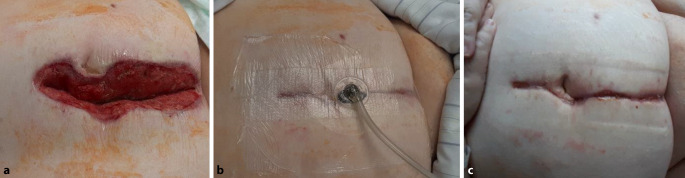
Transparent negative-pressure dressing (TNPD) used for wound closure via secondary intention after conditioning of the wound with a conventional negative-pressure dressing using standard open-pore polyurethane foams. **a** Wound appearance after removal of the polyurethane foam dressing. The entire wound is debrided and granulating. **b** Wound appearance after subsequent dressing with a TNPD. The wound edges were adapted using manual compression and the TNPD was applied. Negative pressure was then applied to the dressing via the tubing connector (continuous suction −125 mm Hg). The wound is adapted by the negative pressure and the self-adhesive dressing, similar to a surgical suture. The transparent nature of the dressing means that it does not have to be removed to enable examination of the wound at any time. **c** Wound appearance 1 day after completion of cutaneous negative-pressure therapy (duration 7 days, one dressing change). Wound treatment is continued with a standard superficial dressing

A case study is used to present a new method of wound closure of a wound healing by secondary intention with the aid of a TNPD (Fig. 3, Video 1).

An obese 62-year-old female patient required an emergency median laparotomy with sigmoid resection due to perforated sigmoid diverticulitis. Postoperative complications included a subcutaneous infection of the laparotomy wound. The wound was fully opened except for the closed fascial suture. Negative-pressure therapy with a polyurethane foam and film occlusion was initiated. After 10 days and two dressing changes, the cavernous deep wound had a completely debrided and granulating surface.

A TNPD was used to continue negative-pressure therapy. Manual compression from a lateral orientation approximated the wound edges such that the cavernous wound opening was closed. The TNPD was then affixed and a self-adhesive trackpad provided a tubing connector to the negative-pressure pump. Before attaching the trackpad, a small polyurethane foam cube (V.A.C. Granufoam, KCI USA, Inc., San Antonio, Texas, USA; Suprasorb CNP Wound Foam, Lohmann & Rauscher International GmbH & Co. KG, Rengsdorf, Germany) was placed on the film opening to facilitate suction. A continuous negative pressure of −125 mm Hg was applied using a negative-pressure pump (ACTIV.A.C; KCI USA, Inc., San Antonio, Texas, USA).

By applying negative pressure, the TOF was drawn completely flat onto the skin and wound (compression). At the same time, wound secretions were continuously suctioned off (drainage). The transparency of the TNPD ensured that the entire wound could be completely observed at any time during therapy. It was not necessary to remove the dressing to assess the wound.

The first dressing change took place on the third day. The superficial TNPD was removed painlessly. What had previously been a gaping wound was now fully adapted with no clinical signs of infection. Small regular suction dimples were seen where the wound surface and skin had been in suction contact with the TOF.

Negative-pressure therapy was continued for another 4 days. To this end, a new TNPD was affixed and connected to the negative pressure generator using the technique described. After a total treatment duration of 7 days, cutaneous negative-pressure therapy was stopped. The TNPD was definitively removed. The small suction dimples were again visible on the skin where it had come into contact with the TOF. The wound was non-irritating and exhibited stable adaptation across its entire length. A conventional wet dressing was applied. When the wound was examined the following day, suction changes were no longer visible. The fully documented course of treatment is shown in the video.

## Discussion

The TNPD is a new type of transparent occlusive dressing to which negative pressure can be applied.

The wound dressing described here consists of an open-pore double-layer drainage film (TOF). The TOF was originally developed for intra-abdominal negative-pressure therapy [4, 5]. The drainage film consists of two transparent membranes that have innumerable perforations that are uniformly arranged and evenly spaced. There is a space between the two membranes that does not collapse when suction is applied. Fluids and gases can be drained through the pores and within the interstitial space. The absorption area is very large compared to the volume of material used. The negative pressure is generated over the entire surface of the drainage film.

Unlike polyurethane foam, the open-pore drainage material of the TOF can be applied intra-abdominally, in direct contact with peritoneal organs. Recently, our group was able to show that the TOF can also be used for intrathoracic negative-pressure therapy in pleural empyema [6].

The adhesion of the TOF to tissue is not as pronounced as with polyurethane foams. It is known from endoscopic application that adhesion of foam drainage to a granulating wound surface can be so strong that the removal procedure can result in tearing of the sponge and even of the drainage tubes [7, 8]. For some years now, we have been using the TOF in endoscopic negative-pressure therapy to produce special thin-lumen film drains, which have considerably expanded the spectrum of endoscopic therapy [9–11]. These film drains are always easy to remove. We have not yet observed any tears in film material in a wide range of applications.

As an open-pore drainage material, the TOF can also be used to provide negative-pressure therapy for wounds on the surface of the body. Because the dressing is less adherent to the tissue surface, it is virtually painless to remove during dressing changes. The debriding properties are less pronounced than those of a polyurethane foam. Also, these observations are already known to us from numerous applications in endoscopic negative-pressure therapy.

With the TNPD assembly shown, the TOF can be used for all types of skin wounds where cutaneous negative-pressure therapy is indicated. The TNPD can also be used to continue and complement negative-pressure therapy with polyurethane foams, in combination with polyurethane foams and together with abdominal negative-pressure therapy. There is a wide range of possible applications. The dressing is easy to handle and to prepare.

In negative-pressure therapy, the available materials must always be used creatively. The size and proportions of the drainage material must be adjusted. The TNPD has been developed from clinical application. Together with industry partners, solutions must be found to provide users with standardized and approved medical devices that also meet the formal requirements for the safety of medical devices.

The video shows a case study demonstrating how the TNPD can be used as an alternative method of wound closure for a wound healing by secondary intention. This was preceded by negative-pressure therapy with polyurethane foams. The major advantage of the TNPD is that it does not require surgical closure with sutures. This innovation in the use of negative-pressure therapy is described here for the first time.

No complications were observed during cutaneous therapy. It is conceivable that infection of the closed subcutaneous tissue may occur, as is the case with surgical wound closure. This would require reopening of the wound and expansion of local treatment. As with any open-pore drainage material, the pores of the TOF can become clogged, rendering the TNPD non-functional. Leakage of the dressing also leads to non-functionality, as negative pressure is no longer exerted on the wound. Both of these potential faults can easily be checked by inspecting the dressing visually or by means of a leakage alarm on the negative-pressure pump, and can be rectified with a dressing change. However, it is arguably a disadvantage to continue negative-pressure therapy whereby the patient is “tethered” to a dressing assembly that involves a drainage tube and pump, as this could lead to prolonged immobility. By contrast, practical clinical experience from numerous applications since 2017 has shown that patient mobility is minimally restricted by a TNPD and acceptance of the dressing is very high. One possibility is the use of smaller pumps that increase mobility and patient comfort. Similarly, it is conceivable that treatment could be continued in an outpatient setting after initial application. This would reduce inpatient treatment times and free up surgical capacity that would otherwise be taken up by surgical wound closure using sutures.

The key advantage of the TNPD over all other cutaneous negative-pressure dressings is its transparency. The wound surface and environment can be inspected visually at any time while simultaneously applying negative pressure, and without the need to remove the dressing.

## Conclusion

Transparent negative-pressure dressing is a new type of see-through wound dressing to which negative pressure can be applied. Thanks to its transparency, the wound can be fully inspected visually during treatment. The dressing is suitable for cutaneous wounds where negative-pressure therapy may be used. The dressing assembly can be used to achieve secondary wound closure.

## Supplementary Information

**Video 1.** The video shows how to make the transparent negative-pressure dressing (TNPD). Furthermore, the treatment cycle of the clinical case study is demonstrated with the application of the TNPD for wound closure of a wound that is healing by secondary intention.

## Abbreviations

NP: Negative pressure
OC: Transparent self-adhesive occlusion film
P: Opening
T: Trackpad
TNPD: Transparent negative-pressure dressing
TOF: Transparent open-pore double-layer drainage film

## Declarations

For this article no studies with human participants or animals were performed by any of the authors. All studies mentioned were in accordance with the ethical standards indicated in each case.
